# County-level socioeconomic status, rurality, and second primary cancer risk among breast cancer survivors in the United States

**DOI:** 10.1007/s10552-025-02054-8

**Published:** 2025-09-04

**Authors:** Katherine Ho, Carolyn Brandt, Jessica Li, Geetanjali Datta, Justin X. Moore, Lene H. S. Veiga, Gretchen L. Gierach, Amy Berrington de González, Jacqueline B. Vo, Cody Ramin

**Affiliations:** 1 Division of Population Sciences, Cedars-Sinai Cancer Center, 6500 Wilshire Blvd, Los Angeles, CA 90048 USA; 2 Department of Epidemiology, Johns Hopkins Bloomberg School of Public Health, Baltimore, MD USA; 3 Division of Cancer Epidemiology and Genetics, National Cancer Institute, Bethesda, MD USA; 4 Community Impact Office, Markey Cancer Center, University of Kentucky, Lexington, KY USA; 5 Center for Health, Engagement, and Transformation, Department of Behavioral Science, University of Kentucky, Lexington, KY USA; 6 Division of Genetics and Epidemiology, The Institute of Cancer Research, London, UK

**Keywords:** Rurality, Socioeconomic status, Second primary cancer, Breast cancer, Survivorship

## Abstract

**Background:**

Breast cancer survivors have an increased risk of second primary cancers (SPCs), the role of county-level socioeconomic status and rurality—factors that may influence access to treatment, surveillance, and preventive care—remains understudied.

**Methods:**

We identified 721,957 women with localized/regional first primary breast cancer who survived ≥ 1 year in 17 Surveillance, Epidemiology, and End Results registries (2000–2018). We used Cox regression to assess associations between county-level median household income (proxy for socioeconomic status), rurality, and their joint effects on invasive SPC risk, adjusting for demographic and clinical factors. We examined risk for all SPCs, non-breast SPCs, and the three most common SPC sites (breast, lung/bronchus, colorectal). Models were further stratified by index breast cancer characteristics.

**Results:**

During 6.1 median years of follow-up, 65,954 survivors developed an SPC (42,400 non-breast; 23,554 breast, 8,338 lung/bronchus, 5,442 colorectal). Survivors from lower-income counties had higher SPC risk (< $50,000 vs. ≥ $75,000: HR = 1.07, 95% CI = 1.04–1.10), driven by lung/bronchus (HR = 1.32, 95% CI = 1.23–1.42) and colorectal cancers (HR = 1.19, 95% CI = 1.09–1.31). Lung/bronchus cancer risk was stronger among younger (age < 50: HR = 1.95, 95% CI = 1.59–2.39, age ≥ 50: HR = 1.20, 95% CI = 1.12–1.28; *p* interaction < 0.001) and Estrogen Receptor (ER)-negative survivors (ER negative: HR = 1.50, 95% CI = 1.31–1.72; ER positive: HR = 1.21, 95% CI = 1.12–1.30; *p* interaction = 0.02). Survivors from rural counties had higher SPC risk compared with most urban counties (HR range:1.07–1.12), especially for lung/bronchus cancer in younger (age < 50: HR = 1.66, 95% CI = 1.34–2.05, age ≥ 50: HR = 1.13, 95% CI = 1.06–1.21; *p* interaction = 0.001) and ER-negative survivors (ER negative: HR = 1.45, 95% CI = 1.26–1.67; ER positive: HR = 1.11, 95% CI = 1.03–1.20; *p* interaction = 0.001). Survivors in rural/lower-income counties had the highest SPC risk compared with urban/higher-income counties (HR-range: 1.20–1.23), particularly for lung/bronchus cancer (HR = 1.57, 95% CI = 1.10–2.23).

**Conclusion:**

Studies are needed to understand factors driving the impact of socioeconomic status and rurality (e.g., access to care) on SPC risk to inform preventive strategies for breast cancer survivors.

**Supplementary Information:**

The online version contains supplementary material available at 10.1007/s10552-025-02054-8.

## Introduction

In the United States, breast cancer mortality has declined by 42% over the past 30 years due to early detection and improved treatment [1–3]. Despite this overall decline, breast cancer survivors have an increased risk of developing a second primary cancer (SPC) [4,5], which is defined as a new malignancy that develops among individuals with a prior initial cancer diagnosis. SPCs can develop synchronously, occurring shortly after the first diagnosis, or metachronously, appearing years later [6]. These cancers are distinct from recurrence or metastasis but may be attributable to treatment-related or shared lifestyle, reproductive, and genetic risk factors with the initial primary cancer [4,7,8].

Notably, there are significant differences in SPC risk by race and ethnicity, with the highest risks among Latina and non-Latina Asian American, Black, and Pacific Islander women [9–12]. These disparities may be partially explained by differences in multilevel factors in accessing cancer treatment and high-quality care, including delays in treatment initiation, higher treatment intensity, barriers in treatment adherence and financial costs, geographic access to oncology care, and post-treatment follow-up and surveillance [9,13–15]. Race and ethnicity, however, are considered social constructs, with disparities rooted in social determinants of health, including socioeconomic status and geographic factors [16–18].

Importantly, socioeconomic status and rurality may independently influence access to cancer treatment and survivorship care, contributing to differences in SPC risk. For example, breast cancer survivors living in lower-income and more rural areas may experience greater challenges in receiving timely and guideline-concordant high-quality treatment, post-treatment surveillance for early detection of SPCs and genetic testing, and preventive care, including behavioral risk factor reduction, which may impact SPC risk [19–22]. Despite this, the impact of county-level socioeconomic status and rurality on SPC risk remains largely understudied. Understanding these disparities among breast cancer survivors may help identify high-risk subgroups and inform the development of targeted interventions to reduce SPC risk.

For this study, we examined the association between county-level socioeconomic status and rurality on SPC risk among a large diverse population-based cohort of breast cancer survivors in the United States. We further assessed these associations by breast cancer characteristics, including age at diagnosis, breast cancer stage, and estrogen receptor (ER) status.

## Methods

### Analytic study population

We included women aged 20–84 who were diagnosed with local or regional first primary breast cancer between 2000 and 2017 (followed through 2018) in the 17 Surveillance, Epidemiology, and End Results (SEER) registries and who survived ≥ 1 year. Breast cancer diagnoses from autopsy or death certificates were excluded. Detailed information on the inclusion criteria is provided in Table S1. Our final analytic population included 721,957 breast cancer survivors. This research was considered exempt from Institutional Review Board (IRB) approval and informed consent by the IRB at Cedars-Sinai Medical Center because the study used deidentified and publicly available data.

### Exposures

County-level median household income and rurality were time dependent and matched to the year of first breast cancer diagnosis. County-level median household income was used as a proxy for socioeconomic status and was defined as household income from the past 12 months, measured in 2019 inflation-adjusted US dollars [23]. Income data were ascertained from the United States 2000 Census or the American Community Survey from 2006 to 2019 and corresponded to the survey year closest to the first breast cancer diagnosis [23]. Median household income was categorized into five pre-defined groups: < $50,000; $50,000–$59,999; $60,000–$69,999; $70,000–$74,999; and ≥ $75,000. Rurality was determined using the rural–urban continuum codes developed by the United States Department of Agriculture, which classify metropolitan areas by population size and nonmetropolitan areas by degree of urbanization and adjacency to metropolitan areas [24,25]. Rural–urban codes were obtained from the United States Census in 2003 and 2013 and categorized into five groups: nonmetropolitan, not adjacent to metropolitan (most rural); nonmetropolitan, adjacent to metropolitan; metropolitan, population < 250,000; metropolitan, population 250,000–1 million; and metropolitan, population > 1 million (most urban). To evaluate the joint association between household income and rurality, we created four combined categories: (1) rural with median household income < $50,000, (2) rural with median household income ≥ $50,000, (3) urban with median household income < $50,000, and (4) urban with median household income ≥ $50,000.

### Outcomes

We defined SPC as an invasive primary cancer diagnosed ≥ 12 months after the first primary breast cancer. Outcomes were combined for all SPC sites (including breast) and all non-breast SPC sites (excluding breast). We also examined the three most common SPC sites: breast, lung, and bronchus, and colorectal cancer. SPC sites were based on the International Classification of Diseases for Oncology (ICD-O-3) site codes. We imposed a 12-month lag period to reduce potential misclassification due to recurrence or metastasis. SEER registries also use strict coding rules for multiple primary cancers, which consider cancer site of origin, date of diagnosis, tumor behavior, and laterality (for paired organs) to reduce potential outcome misclassification. [26]

### Covariates

Demographic and clinical characteristics included age and year at first breast cancer diagnosis, race and ethnicity, stage, histology, ER status, progesterone receptor (PR) status, human epidermal growth factor receptor 2 (HER2) status, and breast cancer subtype (defined as hormone receptor (HR-positive/HER2-negative, HR-positive/HER2-positive, HR-negative/HER2-positive, HR-negative/HER2-negative, or unknown) [27]. HR status was defined as a joint combination of ER and PR status (HR-positive = ER-positive and/or PR-positive; HR-negative = ER-negative and PR-negative). HER2 status and breast cancer subtype are available for women diagnosed with a first primary breast cancer in 2010 or later (*n* = 336,249) and were included in descriptive analyses. Initial treatment included type of surgery, radiotherapy, and chemotherapy. Data for “no” and “unknown” values for radiotherapy and chemotherapy are combined in the SEER registry due to under-ascertainment [28].

### Statistical analysis

Follow-up began 12 months after the date of first primary breast cancer diagnosis and continued until the date of SPC diagnosis, death, loss to follow-up, or end of study (31 December 2018), whichever occurred first. Cox proportional hazards regression was used to estimate hazard ratios (HRs) and 95% confidence intervals (CIs) for the association between county-level median household income, rurality, and their joint effect on the risk of SPC, with analyses conducted for (1) any SPC, (2) any non-breast SPC, and (3) site-specific SPCs including breast, lung and bronchus, and colorectal cancer. Multivariable models adjusted for age at first primary breast cancer diagnosis (< 40, 40–49, 50–59, 60–69, 70 + years), race and ethnicity (Latina, non-Latina American Indian/Alaska Native, non-Latina Asian American, non-Latina Black, non-Latina Pacific Islander, and non-Latina White), year of first breast cancer diagnosis (2000–2004, 2005–2009, 2010–2014, 2015–2017), stage (localized, regional), ER status (positive, negative, borderline/unknown), radiotherapy (yes, no/unknown), and chemotherapy (yes, no/unknown). Schoenfeld residuals and log–log plots were used to test for the proportional hazard assumptions. Since the proportional hazard assumption was violated for radiotherapy, we used stratified Cox models to allow the baseline hazard to vary by strata of radiotherapy. Tests for trend were conducted by modeling median household income as an ordinal variable. We further assessed potential effect modification by index breast cancer characteristics: age at diagnosis (< 50 years, 50 + years; proxy for menopausal status), stage (localized, regional), and ER status (positive, negative). For these analyses, county-level median household income was categorized as < $50,000 and ≥ $50,000, and rurality was examined as nonmetropolitan (rural) and metropolitan (urban). These categories were defined a priori and tests for interaction were estimated with likelihood ratio tests. Analyses were conducted using SEER*Stat software and Stata 17. All statistical tests were two-sided; *p* values < 0.05 were considered statistically significant.

## Results

Among 721,957 breast cancer survivors, the mean age at first breast cancer diagnosis was 58.91 years (SD = 12.64 years), and most survivors were diagnosed with localized disease (66.65%), ER-positive tumors (75.92%), and ductal carcinoma (76.54%) (Table 1). Survivors living in counties with the lowest median household income (< $50,000) were more likely to be non-Latina Black women, diagnosed with an index ER-negative breast cancer, and treated with unilateral mastectomy compared with those in the highest median household income counties (Table S2). Survivors living in the most rural counties (nonmetropolitan, not adjacent to metropolitan) were more likely to be non-Latina White women, slightly older at index diagnosis, and treated with unilateral mastectomy compared to those in the most urban counties (Table S3). Lower-income counties were more likely to be rural/small urban counties, while more rural counties were more likely to also have lower median household income (Tables S2–3). During 6.1 median years of follow-up, there were 65,954 women diagnosed with any SPC and 42,400 were diagnosed with non-breast SPCs. Breast cancer was the most common SPC site (*n* = 23,554; 35.71%), followed by lung and bronchus (*n* = 8,338; 12.64%) and colorectal cancer (*n* = 5,442; 8.25%) (Table S4).

**Table 1:** Distribution of first breast cancer characteristics and county-level factors among 721,957 women diagnosed with a first primary localized/regional breast cancer in 17 SEER registries from 2000 to 2017 and followed through 2018

Index breast cancer characteristics	*n* (%)
Mean age at diagnosis, years (SD)	58.91 (12.64)
*Age at diagnosis, years*	
< 40	43,123 (5.97)
40–49	139,333 (19.30)
50–59	190,676 (26.41)
60–69	184,340 (25.53)
≥ 70	164,485 (22.78)
*Race and ethnicity*	
Latina (all races)	76,377 (10.58)
Non-Latina American Indian/Alaska Native^a^	2974 (0.41)
Non-Latina Asian American	54,853 (7.60)
Non-Latina Black	75,551 (10.47)
Non-Latina Pacific Islander	4491 (0.62)
Non-Latina White	507,711 (70.32)
*Year of diagnosis*	
2000–2004	191,626 (26.54)
2005–2009	194,082 (26.88)
2010–2014	206,617 (28.62)
2015–2017	129,632 (17.96)
*Stage*	
Localized	481,155 (66.65)
Regional	240,802 (33.35)
*Histology*	
Ductal	552,609 (76.54)
Lobular	62,178 (8.61)
Mixed	48,239 (6.68)
Other	58,931 (8.16)
*ER status*	
Positive	548,083 (75.92)
Negative	129,093 (17.88)
Borderline/Unknown	44,781 (6.20)
*PR status*	
Positive	470,764 (65.21)
Negative	198,785 (27.53)
Borderline/Unknown	52,408 (7.26)
*Radiotherapy*	
Yes	394,339 (54.64)
No/Unknown	327,508 (45.36)
*Chemotherapy*	
Yes	310,044 (42.94)
No/Unknown	411,913 (57.06)
*Surgery*	
None	19,628 (2.72)
Breast-conserving surgery	412,761 (57.17)
Unilateral mastectomy	204,171 (28.28)
Bilateral mastectomy	63,027 (8.73)
Unknown	22,370 (3.10)
*HER2 status*^*b*^	
Negative	267,818 (79.65)
Positive	49,442 (14.70)
Borderline/Unknown	18,989 (5.65)
*Breast cancer subtype*^*b*^	
Luminal A (HR + /HER2−)	232,433 (69.13)
Luminal B (HR + /HER2 +)	34,925 (10.39)
HER2 enriched (HR−/HER2 +)	14,379 (4.28)
Triple negative (HR−/HER2−)	35,003 (10.41)
Unknown	19,509 (5.80)
*County-level median household income*	
< $50,000	91,324 (12.65)
50,000—59,999	101,696 (14.09)
60,000—69,999	220,829 (30.59)
70,000—74,999	67,579 (9.36)
≥ $75,000	240,529 (33.32)
*Rurality*	
Nonmetropolitan, not adjacent to metro	30,072 (4.17)
Nonmetropolitan, adjacent to metro	44,812 (6.21)
Metropolitan, < 250,000	53,651 (7.43)
Metropolitan, 250,000–1 million	142,314 (19.71)
Metropolitan, > 1 million	451,108 (62.48)

Values are *n* (%) or mean (SD)

*SEER* Surveillance, Epidemiology, and End Results, *SD* Standard deviation, *ER* Estrogen receptor, *PR* Progesterone receptor, *HER2* Human epidermal growth factor receptor 2, *HR* Hormone receptor

^a^Non-Latina American Indian/Alaska Native: Indian Health Service Purchased/Referred Care Delivery Areas (PRCDA) (*n* = 2,125 (0.29%))

^b^Restricted to women diagnosed with first primary breast cancer in 2010 or later (*n* = 336,249)

### Median household income and SPC risk

**Fig. 1:**
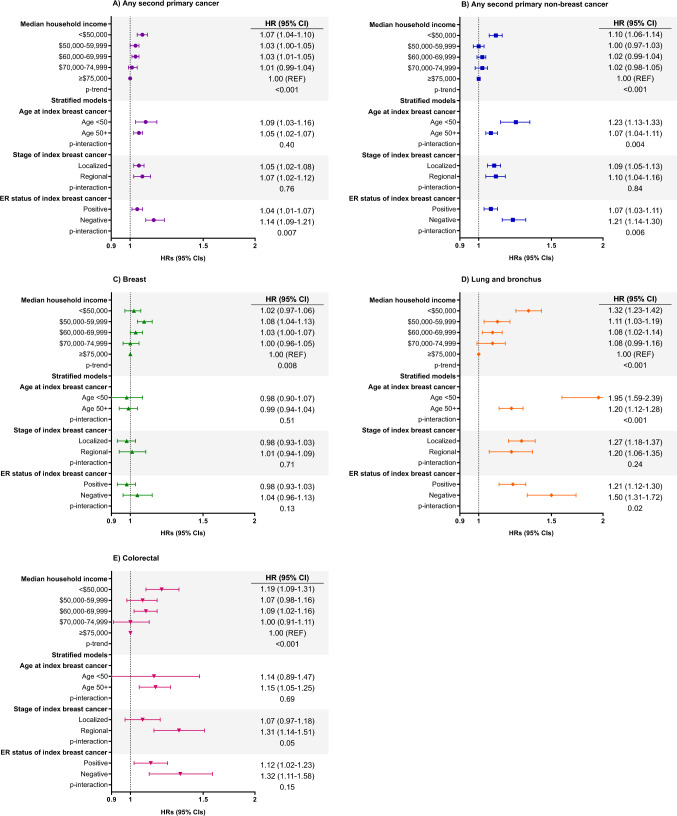
Associations between county-level median household income and second cancer risk among 721,957 women diagnosed with a first primary localized/regional breast cancer in 17 SEER registries from 2000 to 2017, followed through 2018. Models were adjusted for first breast cancer age at diagnosis (< 40, 40–49, 50–59, 60–69, 70 + years), year of diagnosis (2000–2004, 2005–2009, 2010–2014, 2015–2017), ER status (positive, negative, borderline/unknown), race and ethnicity (Latina, non-Latina American Indian/Alaska Native, Asian American, Black, Pacific Islander, White), chemotherapy (yes, no/unknown), and the baseline hazard was stratified by radiation (yes, no/unknown). Stratified models examined the association for median household income dichotomized as < $50,000 vs. ≥ $50,000 (reference)

Compared to women living in counties with the highest median household income (≥ $75,000), women living in lower-income counties generally had an increased risk of any SPC (< $50,000: HR = 1.07, 95% CI = 1.04–1.10; $50,000–$59,999: HR = 1.03, 95% CI = 1.00–1.05; $60,000–$69,999: HR = 1.03, 95% CI = 1.01–1.05; $70,000–$74,999: HR = 1.01, 95% CI = 0.99–1.04; *p* trend < 0.001) (Fig. 1). Results for non-breast SPC were similar but mostly attenuated (< $50,000: HR = 1.10, 95% CI = 1.06–1.14; $50,000–$59,999: HR = 1.00, 95% CI = 0.97–1.03; $60,000–$69,999: HR = 1.02, 95% CI = 0.99–1.04; $70,000–$74,999: HR = 1.02, 95% CI = 0.98–1.05; *p* trend < 0.001) (Fig. 1). An increased risk of SPC among those in the lowest income counties (< $50,000) was driven by the risk of lung and bronchus (HR = 1.32, 95% CI = 1.23–1.42) and colorectal cancer (HR = 1.19, 95% CI = 1.09–1.31) (Fig. 1). For effect modification, we found that the risk of any SPC and non-breast SPC significantly differed by ER status (*p* interactions < 0.05), with higher risk among ER-negative survivors (Fig. 1). Higher risk after ER-negative tumors appeared to be primarily driven by lung and bronchus cancer (ER-positive HR = 1.21, 95% CI = 1.12–1.30; ER-negative HR = 1.50, 9% CI = 1.31–1.72; *p* interaction = 0.02). There were no differences in the risk of any SPCs by age at diagnosis (*p* interaction = 0.40) or stage of diagnosis (*p* interaction = 0.76). In contrast, non-breast SPC risk significantly differed by age, with higher risk among younger women (< 50 years: HR = 1.23, 95% CI = 1.13–1.33; ≥ 50 years: HR = 1.07, 95% CI = 1.04–1.11; *p*-interaction = 0.004). This was largely driven by the risk of second lung and bronchus cancer (< 50 years: HR = 1.95, 95% CI = 1.59–2.39; ≥ 50 years: HR = 1.20, 95% CI = 1.12–1.28; *p* interaction < 0.001).

### Rurality and SPC risk

**Fig. 2:**
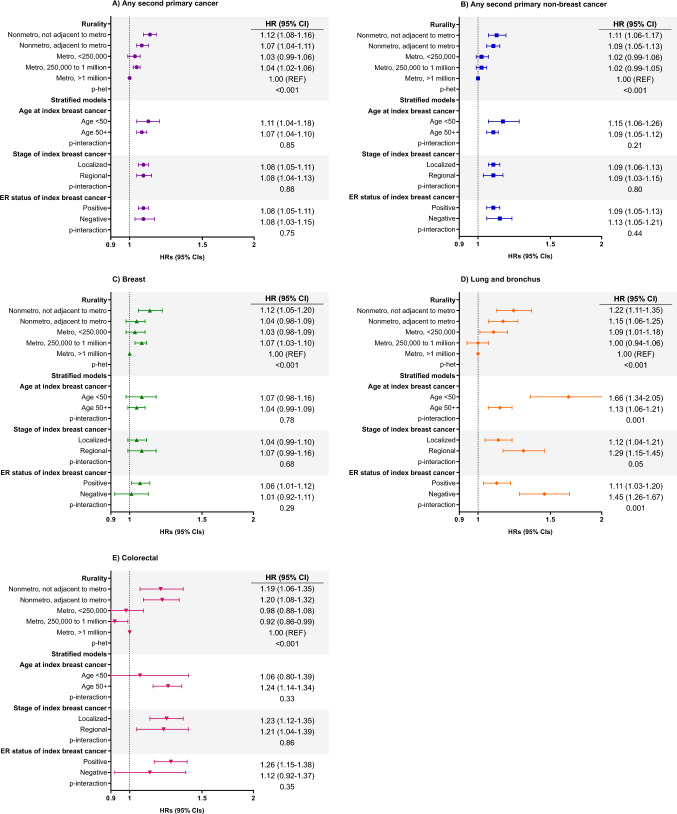
Associations between rurality and second cancer risk among 721,957 women diagnosed with a first primary localized/regional breast cancer in 17 SEER registries from 2000 to 2017, followed through 2018. Models were adjusted for first breast cancer age at diagnosis (< 40, 40–49, 50–59, 60–69, 70 + years), year of diagnosis (2000–2004, 2005–2009, 2010–2014, 2015–2017), ER status (positive, negative, borderline/unknown), race and ethnicity (Latina, non-Latina American Indian/Alaska Native, Asian American, Black, Pacific Islander, White), chemotherapy (yes, no/unknown), and the baseline hazard was stratified by radiation (yes, no/unknown). Stratified models examined the association for rurality dichotomized as rural and urban (reference)

Women living in rural and small to moderately urban counties generally had an increased risk of any SPC and non-breast SPC compared to those in the most urban counties (metropolitan, > 1 million) (Fig. 2). These risks were highest among women living in the most rural counties (any SPC: HR = 1.12, 95% CI = 1.08–1.16; non-breast SPC: HR = 1.11, 95% CI = 1.06 - 1.17), which was driven by risk of breast (HR = 1.12, 95% CI = 1.05–1.20), lung and bronchus (HR = 1.22, 95% CI = 1.11–1.35), and colorectal cancer (HR = 1.19, 95% CI = 1.06–1.35). We found no effect modification by age at diagnosis, stage, or ER status for the risk of any SPC and non-breast SPC overall with respect to rurality (all *p* interactions > 0.05; Fig. 2). However, the association between rurality and the risk of second lung and bronchus cancer significantly differed by age and ER status, with stronger risk among younger women (< 50 years: HR = 1.66, 95% CI = 1.34–2.05; 50 + years: HR = 1.13, 95% CI = 1.06–1.21; *p* interaction = 0.001) or with an ER-negative tumor (ER positive: HR = 1.11, 95% CI = 1.03–1.20; ER negative: HR = 1.45, 95% CI = 1.26–1.67; *p* interaction = 0.001).

### Joint association of median household income and rurality with SPC risk

Survivors living in rural counties, regardless of median household income, and urban counties with the lowest median household income had a significantly increased risk of developing any SPC and non-breast SPC compared to those living in metropolitan counties with higher median household income (Table 2). Risks were highest among those in rural counties with the lowest median household income (any SPC HR = 1.20, 95% CI = 1.03–1.39, non-breast SPC HR = 1.23, 95% CI = 1.03–1.47). These risks appeared to be driven primarily by lung and bronchus cancer and, to some extent, colorectal cancers. There was no significant effect modification for the joint associations of rurality and median household income by age at diagnosis, stage, or ER status of the first breast cancer (Table 2).

**Table 2:** Joint associations for median household income and rurality and risk of second cancer among 721,957 women diagnosed with a first primary localized/regional breast cancer in 17 SEER registries from 2000 to 2017 and followed through 2018^a^

	Any second primary cancer	Any second primary non-breast cancer	Breast	Lung and Bronchus	Colorectal
	HR (95% CI)	HR (95% CI)	HR (95% CI)	HR (95% CI)	HR (95% CI)
Median household income and rurality					
Rural < $50,000	1.20 (1.03 to 1.39)	1.23 (1.03 to 1.47)	1.12 (0.85 to 1.46)	1.57 (1.10 to 2.23)	1.35 (0.85 to 2.14)
Rural ≥ $50,000	1.10 (1.05 to 1.16)	1.11 (1.04 to 1.18)	1.09 (0.99 to 1.18)	1.26 (1.11 to 1.43)	1.34 (1.15 to 1.57)
Urban < $50,000	1.07 (1.04 to 1.10)	1.12 (1.08 to 1.16)	0.99 (0.94 to 1.04)	1.28 (1.19 to 1.38)	1.17 (1.06 to 1.29)
Urban ≥ $50,000	1.00 (REF)	1.00 (REF)	1.00 (REF)	1.00 (REF)	1.00 (REF)
p-het	< 0.001	< 0.001	0.23	< 0.001	0.0001
Age at index breast cancer					
Age < 50					
Rural < $50,000	1.87 (1.37 to 2.54)	2.42 (1.64 to 3.56)	1.35 (0.81 to 2.24)	4.34 (1.79 to 10.49)	2.42 (0.78 to 7.56)
Rural ≥ $50,000	1.11 (0.98 to 1.26)	1.13 (0.95 to 1.36)	1.09 (0.92 to 1.31)	2.02 (1.36 to 3.00)	0.82 (0.42 to 1.58)
Urban < $50,000	1.08 (1.00 to 1.16)	1.20 (1.08 to 1.33)	0.97 (0.88 to 1.08)	1.53 (1.17 to 2.00)	1.17 (0.86 to 1.60)
Urban ≥ $50,000	1.00 (REF)	1.00 (REF)	1.00 (REF)	1.00 (REF)	1.00 (REF)
Age 50 +					
Rural < $50,000	1.08 (0.91 to 1.27)	1.09 (0.90 to 1.33)	1.04 (0.75 to 1.43)	1.39 (0.95 to 2.05)	1.24 (0.74 to 2.06)
Rural ≥ $50,000	1.10 (1.04 to 1.16)	1.11 (1.04 to 1.18)	1.08 (0.97 to 1.19)	1.20 (1.05 to 1.37)	1.39 (1.18 to 1.63)
Urban < $50,000	1.07 (1.04 to 1.10)	1.10 (1.06 to 1.15)	0.99 (0.93 to 1.05)	1.26 (1.17 to 1.37)	1.17 (1.06 to 1.30)
Urban ≥ $50,000	1.00 (REF)	1.00 (REF)	1.00 (REF)	1.00 (REF)	1.00 (REF)
*p* interaction	0.96	0.53	0.99	0.70	0.62
Stage of index breast cancer					
Localized					
Rural < $50,000	1.11 (0.92 to 1.34)	1.16 (0.93 to 1.45)	1.01 (0.71 to 1.43)	1.37 (0.87 to 2.15)	1.47 (0.85 to 2.54)
Rural ≥ $50,000	1.11 (1.04 to 1.18)	1.11 (1.03 to 1.19)	1.10 (0.99 to 1.22)	1.14 (0.97 to 1.33)	1.38 (1.15 to 1.66)
Urban < $50,000	1.07 (1.03 to 1.10)	1.11 (1.07 to 1.16)	0.98 (0.92 to 1.04)	1.30 (1.19 to 1.42)	1.11 (0.99 to 1.25)
Urban ≥ $50,000	1.00 (REF)	1.00 (REF)	1.00 (REF)	1.00 (REF)	1.00 (REF)
Regional					
Rural < $50,000	1.36 (1.07 to 1.73)	1.38 (1.03 to 1.84)	1.33 (0.86 to 2.04)	2.04 (1.15 to 3.60)	1.11 (0.46 to 2.69)
Rural ≥ $50,000	1.09 (1.00 to 1.20)	1.11 (0.99 to 1.24)	1.06 (0.90 to 1.24)	1.56 (1.25 to 1.94)	1.26 (0.94 to 1.68)
Urban < $50,000	1.09 (1.03 to 1.15)	1.12 (1.05 to 1.20)	1.02 (0.93 to 1.12)	1.23 (1.06 to 1.43)	1.31 (1.10 to 1.55)
Urban ≥ $50,000	1.00 (REF)	1.00 (REF)	1.00 (REF)	1.00 (REF)	1.00 (REF)
*p* interaction	0.61	0.82	0.71	0.81	0.03
ER status of index breast cancer					
Positive					
Rural < $50,000	1.12 (0.94 to 1.35)	1.16 (0.93 to 1.43)	1.04 (0.74 to 1.48)	1.47 (0.96 to 2.27)	1.44 (0.85 to 2.44)
Rural ≥ $50,000	1.09 (1.03 to 1.16)	1.07 (0.99 to 1.15)	1.12 (1.01 to 1.24)	1.11 (0.95 to 1.30)	1.34 (1.12 to 1.61)
Urban < $50,000	1.08 (1.04 to 1.11)	1.11 (1.07 to 1.16)	0.99 (0.93 to 1.06)	1.27 (1.16 to 1.39)	1.19 (1.06 to 1.33)
Urban ≥ $50,000	1.00 (REF)	1.00 (REF)	1.00 (REF)	1.00 (REF)	1.00 (REF)
Negative					
Rural < $50,000	1.58 (1.20 to 2.10)	1.73 (1.23 to 2.44)	1.36 (0.83 to 2.22)	1.85 (0.88 to 3.90)	1.20 (0.38 to 3.72)
Rural ≥ $50,000	1.11 (0.99 to 1.25)	1.18 (1.02 to 1.37)	1.00 (0.83 to 1.22)	1.65 (1.26 to 2.17)	1.28 (0.87 to 1.90)
Urban < $50,000	1.13 (1.06 to 1.21)	1.22 (1.13 to 1.33)	1.01 (0.91 to 1.12)	1.50 (1.27 to 1.77)	1.24 (0.99 to 1.56)
Urban ≥ $50,000	1.00 (REF)	1.00 (REF)	1.00 (REF)	1.00 (REF)	1.00 (REF)
*p* interaction	0.13	0.21	0.18	0.99	0.66

*SEER* Surveillance, Epidemiology, and End Results, *REF* Reference group, *HR* Hazard ratio, *CI* Confidence interval, *Het* Heterogeneity

^a^Models were adjusted for age at first breast cancer diagnosis (< 40, 40–49, 50–59, 60–69, 70 + years), year of initial diagnosis (2000–2004, 2005–2009, 2010–2014, 2015–2017), ER status (positive, negative, borderline/unknown), race and ethnicity (Latina, non-Latina American Indian/Alaska Native, Asian American, Black, Pacific Islander, White), chemotherapy (yes, no/unknown), and the baseline hazard was stratified by radiation (yes, no/unknown)

## Discussion

In this large registry-based study of 721,957 breast cancer survivors, we found that women who lived in lower-income or more rural counties generally had a higher risk of developing any SPC and non-breast SPC, primarily driven by the risk of lung and bronchus cancer. The risk of non-breast SPCs, particularly for lung and bronchus, was stronger for those residing in lower-income counties with early-onset breast cancer (aged < 50 years) or for those residing in lower-income or rural counties with ER-negative breast cancer. Overall, the risk of SPC was stronger when considering both county-level income and rurality together, with the highest risks among women living in rural low-income counties and particularly for lung and bronchus cancer. These findings reflect similar socioeconomic and geographic incidence patterns in the general population, which suggests that these structural barriers persist even among those who have survived an initial breast cancer diagnosis. Importantly, these disparities will likely be further exacerbated by the growing number of medical and cancer treatment facility closures in rural areas, and recent changes in Medicare and Medicaid funding, both of which will likely disproportionately impact rural and lower-income breast cancer survivors [29,30].

To our knowledge, no prior studies have examined the association between county-level socioeconomic status, rurality, and SPC risk among breast cancer survivors. However, we have previously observed striking racial and ethnic disparities for SPC risk with higher risk among Non-Latina Black, Non-Latina Asian American/Pacific Islander, and Latina breast cancer survivors compared to their racial and ethnic counterparts in the general population [9]. Adding to this research, our current study provides novel findings on the role of county-level socioeconomic status and rurality as factors, independent of race and ethnicity, that may contribute to disparities in SPC risk. These disparities are likely influenced by limited healthcare access, geographic barriers to oncology and survivorship care, and systemic differences in treatment and post-treatment surveillance and preventive care.

Consistent with prior studies identifying area-level socioeconomic status as an indicator for poorer outcomes along the breast cancer continuum [22,31–35], we found that breast cancer survivors living in lower-income counties had a higher risk of SPC and non-breast SPC, mainly driven by lung and bronchus and colorectal cancer. Lower-income counties are more likely to be medically underserved, which may lead to factors related to increased SPC risk, including barriers to treatment for the index breast cancer, post-treatment surveillance, and preventive strategies during survivorship [33,36–41]. Other factors, such as lack of health insurance, can impose financial toxicities that disproportionately affect patients with lower socioeconomic status and can negatively impact their ability to afford initial and follow-up care [21,42–44]. Importantly, additional upstream factors of social determinants of health may situate minoritized racial and ethnic populations in lower-resourced areas, which may further perpetuate persistent disparities in cancer outcomes [16,17]. Future studies examining the interplay between race, ethnicity, and socioeconomic status are needed to provide insight into how these factors may interact to drive these disparities in SPC risk.

In our study, we further found that women living in more rural counties had an increased risk of SPCs, particularly for lung and bronchus cancer, compared to those in the most urban counties. Prior studies have found that women living in rural areas have lower cancer screening rates, are more likely to be diagnosed at a later stage, and often experience delayed or guideline-discordant care [45–48]. Rurality presents additional challenges related to geographic disparities attributable to larger physical distances to healthcare facilities and fewer healthcare providers, which may lead to poorer healthcare access [49]. Medical oncologists more commonly practice within academic or community settings that are more concentrated in urban areas, creating shortages in the volume of oncology providers practicing in rural locations and higher patient-to-provider ratios [50–52]. Travel distance is also a significant barrier for rural cancer patients as they may have limited access to transportation, greater travel time, and higher cumulative travel costs, which may further exacerbate disparities [19,45,53–55]. These challenges faced by rural breast cancer survivors may impact treatment access and quality (e.g., limited access to advanced radiotherapy techniques that limit chest radiation exposure to normal tissue) and post-treatment surveillance (e.g., access to low-dose CT screening) and preventive care (e.g., resources for smoking cessation) and subsequently lead to an increased risk of SPC, particularly second lung/bronchus cancer. Notably, when examining the joint effect of county-level median household income and rurality, we found that SPC risk, also largely driven by lung and bronchus cancer, was highest among those living in rural counties with the lowest median household income. Future research is warranted to examine the interaction between these interrelated county-level factors on SPC risk with a particular focus on lung and bronchus cancer.

Our study further found that the risk of lung and bronchus cancer was stronger among ER-negative and younger (age < 50 years) breast cancer survivors living in lower-income or rural counties. Given these results, it is possible that genetic predisposition may play a role in the observed stronger risk among younger and ER-negative breast cancer survivors [4,56,57]. Women with early-onset breast cancer are more likely to be diagnosed with aggressive subtypes, including ER-negative tumors, and subsequently receive higher doses of chemotherapy or radiation, which have been linked to increased second SPC risk, including lung cancer [4]. In addition, it is possible that there are shared genetic and environmental factors, including smoking, for ER-negative breast cancer and lung cancer, given the observed mutually increased risk of lung cancer following ER-negative breast cancer and ER-negative breast cancer following lung cancer [58]. Therefore, a focus on early-onset or ER-negative breast cancer survivors and those with a genetic predisposition within socioecological contexts may be informative for understanding social barriers contributing to this increased risk of lung and bronchus cancer, such as lack of genetic testing and differences in cancer treatment and survivorship care. These differences may include lack of preventive strategies, including post-treatment surveillance (e.g., low-dose CT screening) and limited access to smoking cessation resources, including health education and risk reduction counseling.

Strengths of our study include the use of SEER data, which captured a large, diverse, and nationally representative sample of breast cancer survivors with comprehensive cancer characteristics and county-level measures of socioeconomic status and rurality. However, our study is not without limitations. First, counties are relatively large geographic units that may not entirely represent individual-level experiences and exposures. Because it is a larger unit, socioeconomic disadvantage may not be homogeneously distributed throughout the county. Additional studies examining place-based disparities using smaller geographic units such as census tracts or census blocks may provide a more accurate representation of individual- or neighborhood-level exposures [59]. However, county-level measures are still crucial in understanding structural determinants, since decisions regarding healthcare resources and services are often administered at the county level [60,61]. More importantly, county-level measures stress the importance of where someone lives in relation to their cancer outcomes, irrespective of individual socioeconomic status. For instance, high-income individuals may reside in lower-income counties and, therefore, may experience limited resources due to their socioeconomic environment. Second, we were unable to include individual risk factors and health care information such as smoking and health insurance status due to the limitations of registry-based data. Third, the use of a composite variable for socioeconomic status (e.g., Yost index) was not feasible due to data access limitations with time-dependent and individual-level data within the SEER database. Fourth, it is possible that recurrences may have been classified as a second primary breast cancer despite the established coding rules for multiple primary cancers in SEER, which consider cancer site, dates of diagnosis, tumor behavior, and laterality (for paired organs) [26,62]. To reduce this potential misclassification, we excluded any SPCs that developed < 12 months after the first breast cancer. However, future studies that include pathologist classifications and genomic markers to determine true genomic dependence are needed to further refine the classification of multiple primaries and reduce potential misclassification of recurrence as a SPC [62]. Finally, there is possible bias due to missing data for ER status;[63,64] however, this is likely small with only 6% missing ER status in our study.

In summary, our findings demonstrate that breast cancer survivors residing in rural and lower-income counties at diagnosis had an increased risk of SPC, driven largely by lung and bronchus cancer. These results emphasize the importance of examining disparities as a function of where a breast cancer survivor lives at the time of their diagnosis to understand the context of their socioecological environments, which may facilitate or impede both healthcare access and utilization.

## Supplementary Information

Below is the link to the electronic supplementary material.

Supplementary file1 (DOCX 45 KB)

## Data Availability

The data generated in this study is publicly available in the NCI SEER registry database at https://seer.cancer.gov.
