# Supplementary material for: County-level socioeconomic status, rurality, and second primary cancer risk among breast cancer survivors in the United States
Source: Cancer Causes Control. 2025 Sep 4;36(12):1813–24. doi: 10.1007/s10552-025-02054-8 (PMC12630244; doi:10.1007/s10552-025-02054-8)
Supplement: Supplementary file 1 — Supplementary file1 (DOCX 45 KB) [file 10552_2025_2054_MOESM1_ESM.docx]

| Supplemental Table 1. Selection process for 721,957 women diagnosed with a first primary localized/regional breast cancer between 2000-2017 and (followed through 2018) in 17 SEER registries | | |
| --- | --- | --- |
| Number of total records | 8,658,035 | |
| Inclusion criteria | Number of individuals excluded in each step | Remaining number of individuals after exclusion |
| Individuals who survived their first primary cancer (sequence number 0 or 1) | 1,828,890 | 6,829,145 |
| Number of individuals with invasive cancer | 500,711 | 6,328,434 |
| Individuals who were not identified with death certificate or autopsy only | 82,088 | 6,246,346 |
| Individuals with known age records | 537 | 6,245,809 |
| Women with first primary localized/regional breast cancer diagnosed at age 20-84 between 2000-2017 and known county-level attributes | 5,474,285 | 771,524 |
| Women with a date of last contact on index record within latency exclusion period | 21,796 | 749,728 |
| Women with an exit point prior to their entry point | 24,112 | 725,616 |
| Women with unknown race | 3,659 | 721,957 |
| Abbreviations: SEER – Surveillance, Epidemiology and End Results | | |

| Supplemental Table 2. Distribution of first breast cancer characteristics and county-level factors among 721,957 women diagnosed with a first primary localized/regional breast cancer in 17 SEER registries from 2000-2017 and followed through 2018 by median household income | | | | | |
| --- | --- | --- | --- | --- | --- |
|  | <$50,000 | $50,000-59,999 | $60,000-69,999 | $70,000-74,999 | ≥$75,000 |
|  | n = 91324 | n = 101696 | n = 220829 | n = 67579 | n = 240529 |
| Mean age at diagnosis, years (SD) | 59.97 (12.47) | 59.64 (12.55) | 58.81 (12.69) | 58.82 (12.79) | 58.33 (12.63) |
| Age at diagnosis, years |  |  |  |  |  |
| <40 | 4877 (5.34) | 5589 (5.50) | 13748 (6.23) | 4088 (6.05) | 14821 (6.16) |
| 40-<50 | 15045 (16.47) | 17606 (17.31) | 42691 (19.33) | 13581 (20.10) | 50410 (20.96) |
| 50-<60 | 23469 (25.70) | 26546 (26.10) | 58072 (26.30) | 17699 (26.19) | 64890 (26.98) |
| 60-<70 | 25410 (27.82) | 27351 (26.89) | 56513 (25.59) | 16559 (24.50) | 58507 (24.32) |
| ≥70 | 22523 (24.66) | 24604 (24.19) | 49805 (22.55) | 15652 (23.16) | 51901 (21.58) |
| Race and ethnicity |  |  |  |  |  |
| Latina (all races) | 5441 (5.96) | 9870 (9.71) | 34426 (15.59) | 6307 (9.33) | 20333 (8.45) |
| Non-Latina American Indian/Alaska Native^a^ | 608 (0.67) | 753 (0.74) | 725 (0.33) | 230 (0.34) | 658 (0.27) |
| Non-Latina Asian | 805 (0.88) | 2349 (2.31) | 18326 (8.30) | 3318 (4.91) | 30055 (12.50) |
| Non-Latina Black | 19001 (20.81) | 12134 (11.93) | 24175 (10.95) | 5142 (7.61) | 15099 (6.28) |
| Non-Latina Pacific Islander | 57 (0.06) | 277 (0.27) | 1034 (0.47) | 360 (0.53) | 2763 (1.15) |
| Non-Latina White | 65412 (71.63) | 76313 (75.04) | 142143 (64.37) | 52222 (77.28) | 171621 (71.35) |
| Year of diagnosis |  |  |  |  |  |
| 2000-2004 | 16790 (18.39) | 22506 (22.13) | 57000 (25.81) | 22341 (33.06) | 72989 (30.35) |
| 2005-2009 | 24112 (26.40) | 25865 (25.43) | 59244 (26.83) | 20692 (30.62) | 64169 (26.68) |
| 2010-2014 | 32838 (35.96) | 35360 (34.77) | 65666 (29.74) | 15530 (22.98) | 57223 (23.79) |
| 2015-2017 | 17584 (19.25) | 17965 (17.67) | 38919 (17.62) | 9016 (13.34) | 46148 (19.19) |
| Stage |  |  |  |  |  |
| Localized | 59928 (65.62) | 67568 (66.44) | 145484 (65.88) | 45223 (66.92) | 162952 (67.75) |
| Regional | 31396 (34.38) | 34128 (33.56) | 75345 (34.12) | 22356 (33.08) | 77577 (32.25) |
| Histology |  |  |  |  |  |
| Ductal | 71409 (78.19) | 79746 (78.42) | 167459 (75.83) | 50301 (74.43) | 183694 (76.37) |
| Lobular | 7403 (8.11) | 8543 (8.40) | 18337 (8.30) | 6066 (8.98) | 21829 (9.08) |
| Mixed | 3922 (4.29) | 5037 (4.95) | 16457 (7.45) | 5692 (8.42) | 17131 (7.12) |
| Other | 8590 (9.41) | 8370 (8.23) | 18576 (8.41) | 5520 (8.17) | 17875 (7.43) |
| ER status |  |  |  |  |  |
| Positive | 66274 (72.57) | 76752 (75.47) | 166120 (75.23) | 52272 (77.35) | 186665 (77.61) |
| Negative | 18648 (20.42) | 18712 (18.40) | 40081 (18.15) | 11860 (17.55) | 39792 (16.54) |
| Borderline/Unknown | 6402 (7.01) | 6232 (6.13) | 14628 (6.62) | 3447 (5.10) | 14072 (5.85) |
| PR status |  |  |  |  |  |
| Positive | 57684 (63.16) | 66234 (65.13) | 141134 (63.91) | 45195 (66.88) | 160517 (66.73) |
| Negative | 26782 (29.33) | 28615 (28.14) | 61207 (27.72) | 18553 (27.45) | 63628 (26.45) |
| Borderline/Unknown | 6,858 (7.51) | 6,847 (6.73) | 18,488 (8.37) | 3,831 (5.67) | 16,384 (6.81) |
| Radiotherapy |  |  |  |  |  |
| Yes | 47468 (51.98) | 55982 (55.05) | 111832 (50.64) | 38917 (57.59) | 140250 (58.31) |
| No/unknown | 43856 (48.02) | 45714 (44.95) | 108997 (49.36) | 28662 (42.41) | 100279 (41.69) |
| Chemotherapy |  |  |  |  |  |
| Yes | 41405 (45.34) | 44330 (43.59) | 92482 (41.88) | 29143 (43.12) | 102684 (42.69) |
| No/unknown | 49919 (54.66) | 57366 (56.41) | 128347 (58.12) | 38436 (56.88) | 137845 (57.31) |
| Surgery |  |  |  |  |  |
| None | 2498 (2.74) | 2493 (2.45) | 6968 (3.16) | 1660 (2.46) | 6009 (2.50) |
| Breast conserving surgery | 46415 (50.82) | 55184 (54.26) | 124008 (56.16) | 40113 (59.36) | 147041 (61.13) |
| Unilateral mastectomy | 31036 (33.98) | 30708 (30.20) | 63398 (28.71) | 18230 (26.98) | 60799 (25.28) |
| Bilateral mastectomy | 7920 (8.67) | 10160 (9.99) | 19823 (8.98) | 5728 (8.48) | 19396 (8.06) |
| Unknown | 3455 (3.78) | 3151 (3.10) | 6632 (3.00) | 1848 (2.73) | 7284 (3.03) |
| HER2 status^b^ |  |  |  |  |  |
| Negative | 39499 (78.34) | 42701 (80.08) | 82434 (78.82) | 19841 (80.83) | 83343 (80.63) |
| Positive | 7718 (15.31) | 7670 (14.38) | 15406 (14.73) | 3562 (14.51) | 15086 (14.59) |
| Borderline/unknown | 3205 (6.36) | 2954 (5.54) | 6745 (6.45) | 1143 (4.66) | 4942 (4.78) |
| Breast cancer subtype^b^ |  |  |  |  |  |
| Luminal A (HR+/HER2-) | 32943 (65.33) | 36634 (68.70) | 71565 (68.43) | 17391 (70.85) | 73900 (71.49) |
| Luminal B (HR+/HER2+) | 5468 (10.84) | 5303 (9.94) | 10849 (10.37) | 2569 (10.47) | 10736 (10.39) |
| HER2 enriched (HR-/HER2+) | 2223 (4.41) | 2347 (4.40) | 4514 (4.32) | 979 (3.99) | 4316 (4.18) |
| Triple negative (HR-/HER2-) | 6492 (12.88) | 6021 (11.29) | 10741 (10.27) | 2409 (9.81) | 9340 (9.04) |
| Unknown | 3296 (6.54) | 3020 (5.66) | 6916 (6.61) | 1198 (4.70) | 5079 (4.91) |
| Rural urban continuum |  |  |  |  |  |
| Nonmetropolitan, not adjacent to metropolitan | 19432 (21.28) | 7600 (7.47) | 2126 (0.96) | 278 (0.41) | 636 (0.26) |
| Nonmetropolitan, adjacent to metropolitan | 25117 (27.50) | 11226 (11.04) | 5539 (2.51) | 474 (0.70) | 2456 (1.02) |
| Metropolitan, <250,000 | 16936 (18.54) | 21620 (21.26) | 10233 (4.63) | 2814 (4.16) | 2048 (0.85) |
| Metropolitan, 250,000-1 million | 12977 (14.21) | 35859 (35.26) | 30804 (13.95) | 19784 (29.28) | 42890 (17.83) |
| Metropolitan, >1 million | 16862 (18.46) | 25391 (24.97) | 172127 (77.95) | 44229 (65.45) | 192499 (80.03) |
| Note. Values are n (%) or mean (SD)  Abbreviations: SEER – Surveillance, Epidemiology and End Results, SD – Standard deviation, ER – Estrogen receptor, HER2 – Human epidermal growth factor receptor 2, HR – Hormone receptor  ^a^ Restricted to Non-Latina American Indian/Alaska Native: Indian Health Service Purchased/Referred Care Delivery Areas (PRCDA) (<$50,000: n=525 (0.57%); $50,000 - 59,999: n=638 (0.63%); $60,000 - 69,999: n=451 (0.20%); $70,000 - 74,999: n=174 (0.26%); ≥$75,000: n=337 (0.14%))  ^b^ Restricted to women diagnosed with first primary breast cancer in 2010 or later (n=336,249) | | | | | |

| Supplemental Table 3. Distribution of first breast cancer characteristics and county-level factors among 721,957 women diagnosed with a first primary breast cancer in primary localized/regional breast cancer in 17 SEER registries from 2000-2017 and followed through 2018 by rurality | | | | | |
| --- | --- | --- | --- | --- | --- |
|  | Nonmetropolitan, not adjacent to metropolitan | Nonmetropolitan, adjacent to metropolitan | Metropolitan, <250,000 | Metropolitan, 250,000-1 million | Metropolitan, >1 million |
|  | n = 30072 | n = 44812 | n = 53651 | n = 142314 | n = 451108 |
| Mean age at diagnosis, years (SD) | 60.72 (12.36) | 60.50 (12.39) | 59.82 (12.58) | 59.14 (12.61) | 58.46 (12.68) |
| Age at diagnosis, years |  |  |  |  |  |
| <40 | 1398 (4.65) | 2117 (4.72) | 2877 (5.36) | 8275 (5.81) | 28456 (6.31) |
| 40-<50 | 4613 (15.34) | 7210 (16.09) | 9246 (17.23) | 26553 (18.66) | 91711 (20.33) |
| 50-<60 | 7608 (25.30) | 11172 (24.93) | 13799 (25.72) | 37509 (26.36) | 120588 (26.73) |
| 60-<70 | 8415 (27.98) | 12560 (28.03) | 14461 (26.95) | 36944 (25.96) | 111960 (24.82) |
| ≥70 | 8038 (26.73) | 11753 (26.23) | 13268 (24.73) | 33033 (23.21) | 98393 (21.81) |
| Race and ethnicity |  |  |  |  |  |
| Latina (all races) | 1290 (4.29) | 1443 (3.22) | 4052 (7.55) | 15444 (10.85) | 54148 (12.00) |
| Non-Latina American Indian/Alaska Native^a^ | 346 (1.15) | 331 (0.74) | 471 (0.88) | 630 (0.44) | 1196 (0.27) |
| Non-Latina Asian | 1029 (3.42) | 246 (0.55) | 1059 (1.97) | 9945 (6.99) | 42574 (9.44) |
| Non-Latina Black | 1372 (4.56) | 4618 (10.31) | 5402 (10.07) | 12505 (8.79) | 51654 (11.45) |
| Non-Latina Pacific Islander | 627 (2.08) | 21 (0.05) | 253 (0.47) | 2113 (1.48) | 1477 (0.33) |
| Non-Latina White | 25408 (84.49) | 38153 (85.14) | 42414 (79.06) | 101677 (71.45) | 300059 (66.52) |
| Year of diagnosis |  |  |  |  |  |
| 2000-2004 | 8154 (27.11) | 13117 (29.27) | 15697 (29.26) | 35819 (25.17) | 118839 (26.34) |
| 2005-2009 | 8052 (26.78) | 12448 (27.78) | 14891 (27.76) | 37464 (26.32) | 121227 (26.87) |
| 2010-2014 | 8607 (28.62) | 11754 (26.23) | 13990 (26.08) | 42482 (29.85) | 129784 (28.77) |
| 2015-2017 | 5259 (17.49) | 7493 (16.72) | 9073 (16.91) | 26549 (18.66) | 81258 (18.01) |
| Stage |  |  |  |  |  |
| Localized | 20284 (67.45) | 30068 (67.10) | 35568 (66.30) | 95838 (67.34) | 299397 (66.37) |
| Regional | 9788 (32.55) | 14744 (32.90) | 18083 (33.70) | 46476 (32.66) | 151711 (33.63) |
| Histology |  |  |  |  |  |
| Ductal | 23160 (77.02) | 34751 (77.55) | 41977 (78.24) | 110790 (77.85) | 341931 (75.80) |
| Lobular | 2410 (8.01) | 3712 (8.28) | 4485 (8.36) | 12441 (8.74) | 39130 (8.67) |
| Mixed | 1515 (5.04) | 2151 (4.80) | 2575 (4.80) | 7795 (5.48) | 34203 (7.58) |
| Other | 2987 (9.93) | 4198 (9.37) | 4614 (8.60) | 11288 (7.93) | 35844 (7.95) |
| ER status |  |  |  |  |  |
| Positive | 22364 (74.37) | 32577 (72.70) | 40212 (74.95) | 108388 (76.16) | 344542 (76.38) |
| Negative | 5479 (18.22) | 8429 (18.81) | 9885 (18.42) | 25761 (18.10) | 79539 (17.63) |
| Borderline/Unknown | 2229 (7.41) | 3806 (8.49) | 3554 (6.62) | 8165 (5.74) | 27027 (5.99) |
| PR status |  |  |  |  |  |
| Positive | 19,549 (65.01) | 28,362 (63.29) | 34,700 (64.68) | 93,600 (65.77) | 294,553 (65.30) |
| Negative | 8,139 (27.07) | 12,439 (27.76) | 15,130 (28.20) | 39,432 (27.71) | 123,645 (27.41) |
| Borderline/Unknown | 2,384 (7.93) | 4,011 (8.95) | 3,821 (7.12) | 9,282 (6.52) | 32,910 (7.30) |
| Radiotherapy |  |  |  |  |  |
| Yes | 15147 (50.37) | 22597 (50.43) | 28892 (53.85) | 81042 (56.95) | 246771 (54.70) |
| No/unknown | 14925 (49.63) | 22215(49.57) | 24759 (46.15) | 61272 (43.05) | 204337 (45.30) |
| Chemotherapy |  |  |  |  |  |
| Yes | 12747 (42.39) | 18895 (42.17) | 22864 (42.62) | 60779 (42.71) | 194759 (43.17) |
| No/unknown | 17325 (57.61) | 25917 (57.83) | 30787 (57.38) | 81535 (57.29) | 256349 (56.83) |
| Surgery |  |  |  |  |  |
| None | 640 (2.13) | 1064 (2.37) | 1270 (2.37) | 3405 (2.39) | 13249 (2.94) |
| Breast conserving surgery | 15589 (51.84) | 23071 (51.48) | 29158 (54.35) | 83515 (58.68) | 261428 (57.95) |
| Unilateral mastectomy | 10396 (34.57) | 15093 (33.68) | 16591 (30.92) | 39521 (27.77) | 122570 (27.17) |
| Bilateral mastectomy | 2562 (8.52) | 3852 (8.60) | 4402 (8.20) | 11960 (8.40) | 40251 (8.92) |
| Unknown | 885 (2.94) | 1732 (3.87) | 2230 (4.16) | 3913 (2.75) | 13610 (3.02) |
| HER2 status^b^ |  |  |  |  |  |
| Negative | 10927 (78.80) | 15331 (79.65) | 18529 (80.34) | 55456 (80.33) | 167575 (79.40) |
| Positive | 1998 (14.41) | 2713 (14.10) | 3361 (14.57) | 10009 (14.50) | 31361 (14.86) |
| Borderline/unknown | 941 (6.79) | 1203 (6.25) | 1173 (5.09) | 3566 (5.17) | 12106 (5.74) |
| Breast cancer subtype^b^ |  |  |  |  |  |
| Luminal A (HR+/HER2-) | 9446 (68.12) | 13053 (67.82) | 15888 (68.89) | 48102 (69.68) | 145944 (69.15) |
| Luminal B (HR+/HER2+) | 1400 (10.10) | 1919 (9.97) | 2371 (10.28) | 7015 (10.16) | 22220 (10.53) |
| HER2 enriched (HR-/HER2+) | 589 (4.25) | 787 (4.09) | 977 (4.24) | 2968 (4.30) | 9058 (4.29) |
| Triple negative (HR-/HER2-) | 1453 (10.48) | 2255 (11.72) | 2623 (11.37) | 7297 (10.57) | 21375 (10.13) |
| Unknown | 978 (7.05) | 1233 (6.41) | 1204 (5.22) | 3649 (5.29) | 12445 (5.90) |
| Median household income |  |  |  |  |  |
| <$50,000 | 19432 (64.62) | 25117 (56.05) | 16936 (31.57) | 12977 (9.12) | 16862 (3.74) |
| $50,000 - 59,999 | 7600 (25.27) | 11226 (25.05) | 21620 (40.30) | 35859 (25.20) | 25391 (5.63) |
| $60,000 - 69,999 | 2126 (7.07) | 5539 (12.36) | 10233 (19.07) | 30804 (21.65) | 172127 (38.16) |
| $70,000 - 74,999 | 278 (0.92) | 474 (1.06) | 2814 (5.25) | 19784 (13.90) | 44229 (9.80) |
| ≥$75,000 | 636 (2.11) | 2456 (5.48) | 2048 (3.82) | 42890 (30.14) | 192499 (42.67) |
| Note. Values are n (%) or mean (SD)  Abbreviations: SEER – Surveillance, Epidemiology and End Results, SD – Standard deviation, ER – Estrogen receptor, HER2 – Human epidermal growth factor receptor 2, HR – Hormone receptor  ^a^ Restricted to Non-Latina American Indian/Alaska Native: Indian Health Service Purchased/Referred Care Delivery Areas (PRCDA) (Nonmetropolitan, not adjacent to metropolitan: n=301 (1.00%); Nonmetropolitan, adjacent to metropolitan: n=311 (0.69%); Metropolitan, <250,000: n=415 (0.77%); Metropolitan, 250,000-1 million: n=412 (0.29%); Metropolitan, >1 million: n=686 (0.15%))  ^b^ Restricted to women diagnosed with first primary breast cancer in 2010 or later (n=336,249) | | | | | |

| Supplemental Table 4. Distribution of second cancer sites for second primary cancer and second primary non-breast cancers among 721,957 women diagnosed with a first primary localized/regional breast cancer in 17 SEER registries from 2000-2017 and followed through 2018 | | |
| --- | --- | --- |
| Second cancer site | Second primary cancer  (n=65,954) | Second primary non-breast cancer  (n=42,400) |
|  | n (%) | n (%) |
| Oral cavity and pharynx | 1,033 (1.57) | 1,033 (2.44) |
| Digestive system |  |  |
| Esophagus | 289 (0.44) | 289 (0.68) |
| Stomach | 806 (1.22) | 806 (1.90) |
| Small intestine | 299 (0.45) | 299 (0.71) |
| Colon and rectum | 5,442 (8.25) | 5,442 (12.83) |
| Liver and intrahepatic bile duct | 3,425 (5.19) | 3,425 (8.08) |
| Respiratory system |  |  |
| Lung and bronchus | 8,338 (12.64) | 8,338 (19.67) |
| Other respiratory | 230 (0.35) | 230 (0.54) |
| Bones and joints | 69 (0.10) | 69 (0.16) |
| Soft tissue including heart | 533 (0.81) | 533 (1.26) |
| Skin excluding basal and squamous |  |  |
| Melanoma | 2,324 (3.52) | 2,324 (5.48) |
| Other non-epithelial skin | 287 (0.44) | 287 (0.68) |
| Breast | 23,554 (35.71) | -- |
| Female genital system |  |  |
| Cervix uteri | 351 (0.53) | 351 (0.83) |
| Corpus and Uterus, NOS | 4,128 (6.26) | 4,128 (9.74) |
| Ovary | 1,652 (2.50) | 1,652 (3.90) |
| Other female genital system | 632 (0.96) | 632 (1.49) |
| Urinary system |  |  |
| Urinary bladder | 1,386 (2.10) | 1,386 (3.27) |
| Kidney and renal pelvis | 1,490 (2.26) | 1,490 (3.51) |
| Other urinary organs | 102 (0.15) | 102 (0.24) |
| Eye and orbit | 106 (0.16) | 106 (0.25) |
| Brain and other nervous system | 469 (0.71) | 469 (1.11) |
| Endocrine system |  |  |
| Thyroid | 1,900 (2.88) | 1,900 (4.48) |
| Other endocrine | 55 (0.08) | 55 (0.13) |
| Lymphoma |  |  |
| Hodgkin lymphoma | 88 (0.13) | 88 (0.21) |
| Non-Hodgkin lymphoma | 2,078 (3.15) | 2,078 (4.90) |
| Myeloma | 766 (1.16) | 766 (1.81) |
| Leukemia |  |  |
| Lymphocytic leukemia | 537 (0.81) | 537 (1.27) |
| Myeloid and monocytic leukemia | 1,273 (1.93) | 1,273 (3.00) |
| Other leukemia | 85 (0.13) | 85 (0.20) |
| Mesothelioma | 72 (0.11) | 72 (0.17) |
| Kaposi sarcoma | 11 (0.02) | 11 (0.03) |
| Miscellaneous | 2,144 (3.25) | 2,144 (5.06) |
